# X-ray CT Investigation of Bond Mechanism in Reinforced SCC Elements of Different Placement Technology

**DOI:** 10.3390/ma14216236

**Published:** 2021-10-20

**Authors:** Piotr Dybeł, Milena Kucharska

**Affiliations:** Faculty of Civil Engineering and Resource Management, AGH University of Science and Technology, 30-059 Cracow, Poland; kucharska@agh.edu.pl

**Keywords:** computed tomography, self-compacting concrete, bond mechanism, bottom-up placing

## Abstract

The effect of different placing methods of the self-compacting concrete (SCC) mix—from the top and from the bottom of the form—on the bond failure mechanism was investigated within the scope of this paper. Existing studies regarding the known mechanisms of bond failure do not consider the bottom-up method of concrete placing, which improves the quality of the concrete microstructure around reinforcing bars. Background tests were performed on panel elements with dimensions of 800 × 480 × 160 mm. Ribbed steel reinforcing bars with a diameter of 16 mm were used in the tests, which were placed horizontally in the forms. A pull-out method was used to investigate the bond strength. X-ray computed tomography (CT) was used as a novel and non-destructive technique that allowed a 3D insight into the bond between the rebar and the concrete after the ultimate bond stress had been reached. The results provided a clear description of the phenomena occurring during the fresh state of concrete in the vicinity of rebars (bleeding, plastic settlement, vertical density variation) and showed their significance for bond mechanisms. Finally, it was demonstrated that placing the mix from the bottom of the form resulted in the same bond failure mechanism for both bars located at the top and the bottom of the panel elements. This was translated into identical bond properties throughout the element with regard to bond stiffness and bond strength. It was found that the described and known mechanisms of bond failure are only an idealized description of the performance of the reinforcing bar-concrete joint. The analysis of the steel–concrete interface (SCI) imaging indicated that, in reality, the forming bond failure mechanisms were a complex process that could be affected by many factors.

## 1. Introduction

Concrete is a material that, despite its widespread use, undergoes constant modification and is adapted to the needs of architects, constructors and the environment. In consequence, self-compacting concrete (SCC) was developed. Compared to traditional concretes, SCCs exhibit special rheological properties that ensure the gravity filling of a form or formwork of any shape without the occurrence of segregation and with no additional mechanical compaction [1,2] while maintaining a seamless surface finish. By modifying the composition of the mix and its rheological properties, the interaction conditions between hardened concrete and reinforcing bars are also adjusted.

The bond between reinforced steel and hardened concrete is paramount for the homogeneity of the structure of the final composition [3]. Aside from the individual properties of steel and concrete, the consolidation of the fresh concrete mix before solidification has also been found to be an important factor determining bond strength. Filling the formwork from above induces the occurrence of zones in the bottom parts, where compacting conditions are better than those in the upper regions. This effect is associated with fresh concrete consolidation. In this process, water trickles upwards (the phenomenon of bleeding) while concrete sinks towards the bottom of the mould (plastic settlement). Such processes impair the bond between concrete and horizontally positioned rebars. The bleeding can alter the physicochemical properties, leading to the extension of voids at the reinforcement-concrete contact area and consequently corrosion and the degradation of the bond strength. The ongoing settlement of fresh concrete contributes to the reduction of the effective projection of ribs and, in consequence, of the bond strength. The literature describes this phenomenon as the top-bar effect and defines it as bond strength decline in relation to the depth of the concrete underlying horizontal rebars [4,5,6]. Thus, proper stability is paramount in deep structural elements.

The unique rheological properties of the self-compacting mix provide the possibility of placing it in other variants than the traditional mix. The formwork or a form may also be filled from the bottom [7]. The bottom-up placing method is executed by pumping the mixture through a specially designed valve that is permanently placed in the formwork system. This method is most often used for vertical elements such as walls or columns with complex geometry or dense reinforcement or that are difficult access from above [8].

No comprehensive research has yet been conducted into the effect of the bottom-up placing of the self-compacting concrete mixture on its bonding to steel rebars; neither is this technology considered in the standard guidelines. Preliminary studies were presented in [9,10]. It was observed that, in general, the bottom-up placing of the concrete mixture improved the quality of the hardened concrete in terms of the bond strength and properties of bond stiffness, bond strength, top-bar effect, and the proximity of rebar to the casting point effect in the upper regions of the element. The hypothesis assumes that the bottom-up placing of the mix reduces air voids and pores in the reinforcing bar–concrete contact zone, which contributes to an improvement in the quality of concrete cover and thus the bond properties. This phenomenon can be verified by surface image analysis after the splitting of a sample after pull-out test [11] or by videomicroscope analysis [3,6,12]. Another modern and non-destructive method of examining the concrete cover around the reinforcing bar is X-ray computed tomography (X-ray CT). X-ray CT is based on a representation of the three-dimensional structure of the material from multiple 2D tomographic images using X-ray spectroscopy. After image reconstruction, any variation in the material such as density change or presence of pores can be visualized and measured [13,14].

In the literature, issues related to the bond failure mechanism have been widely reported. Generally, bond failure occurs when the concrete fails before the reinforcement reaches its yield strength. Hence, an investigation of the bond mechanism of reinforcing bars is essential due to its direct relationship with the serviceability, shear, and flexural strengths of reinforced concrete (RC) structures [15]. Moreover, in spite of the great research interest in the subject, studies related to SCCs have so far received little attention. Known solutions and analyses of the bond failure mechanism do not consider the variant of thr bottom-up placing of the SCC mixture, which, according to [16], significantly affects the microstructure of the rebar–concrete interface. In view of the discussed problems, the aim of this work is to analyze the influence of placing the self-compacting mixture—from the top and bottom of the form—on the mechanism of bond failure between rebar and SCC. Therefore, X-ray CT was used to image the steel–concrete interface (SCI).

## 2. Bond Phenomenon Description

The bond phenomenon has been investigated, characterized, and analytically modeled at three different scales in the literature. Bond response scales are typically defined by the dimensions of the structural element, the reinforcing bar, and the lugs on the bar. Local bond conditions are generally defined at the scale of the reinforcing bar, which is based on the strength properties of both materials. The bond in a reinforced concrete element is induced by several mechanisms in the vicinity of the concrete–steel interface. This was presented in detail in the literature [3,17,18,19,20,21].

**Figure 1:**
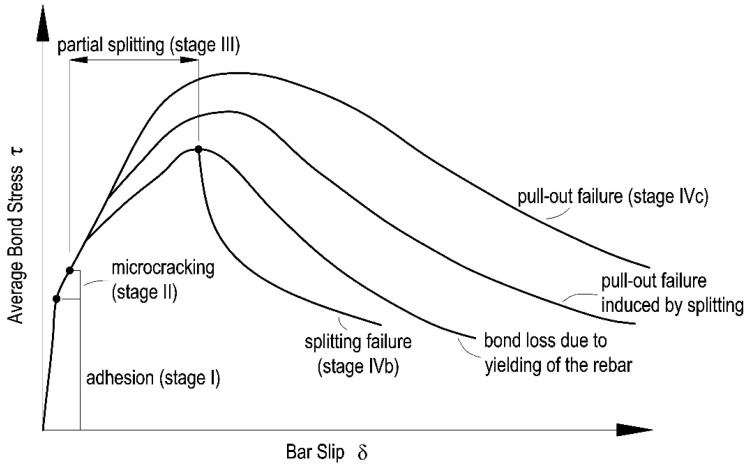
Typical local bond stress–slip relationship in different confinement conditions.

In general, the interaction between concrete and reinforcing bars subjected to a pull-out force is characterized by the following four stages (not all of them occur in the case of plain bars): stage I—uncracked state; stage II—initial cracking; stage III—concrete crushing and development of cracks; stage IVa—the destruction of bonds of plain bars; stage IVb—the destruction of bonds of unconfined ribbed bars or loss of bonds due to yielding of the reinforcement; stage IVc—the destruction of bonds of confined ribbed bars. The stages of the bond phenomenon in ribbed rebars with a distinction between confinement conditions are shown in Figure 1.

**Figure 2:**
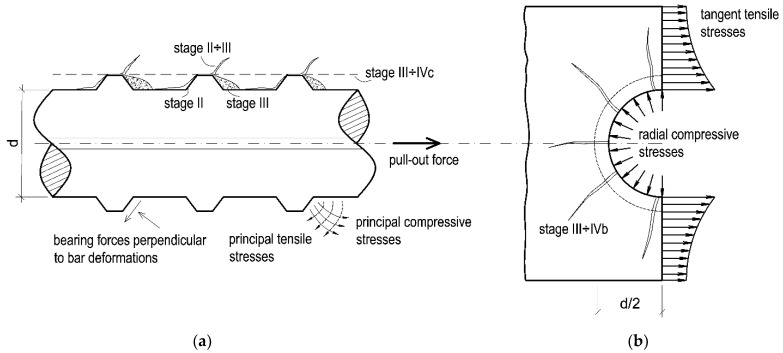
Cracking and damage mechanisms during bond action: (**a**) phenomena in the vicinity of the ribs, (**b**) cross-sectional view between ribs of reinforcement.

Phenomena accompanying the development of the bond mechanism are presented in Figure 2. At first, bond stresses are generated by the chemical adhesion between the steel and concrete cover, which are further accompanied by the micromechanical interaction associated with the roughness of the steel surface (stage I). However, this type of chemical and mechanical adhesion is relatively minor and disappears as soon as a relatively slight slippage of the rebar occurs. At that point, the transfer of forces between pull-out rebar and concrete is ensured by the mechanical engagement between the ribs of the rebar and the surrounding concrete. In this phase, firstly, on the back of the rib near the loading end of the rebar, a tensile microcrack appears, which subsequently propagates towards the opposite direction of a pull-out force, creating a longitudinal slip microcrack on the rebar surface. Subsequently, at the tips of the lugs, the transverse microcrack is formed as an extension of the first crack, allowing the rebar to slip; however, no splitting is noted due to the wedging of the lugs (stage II). As the bond stress increases, the longitudinal cracks (splitting cracks) propagate radially, which is the result of wedging action enhanced by the crushed concrete stuck to the front of the ribs (stage III). After these cracks are formed, the tensile load is mainly transferred by the friction on the periphery and compressions on the ribs of the reinforcement.

The bond failure of ribbed reinforcing bars may take place in either of two distinct modes. The first is associated with the splitting of the concrete cover (stage IVb) and the second with rebar pull-out (stage IVc). If the thickness of the concrete cover is less than three reinforcement diameters, splitting cracks propagate along the radial components of the forces transmitted by the ribs, parallel to the reinforcing bars (Figure 2b), causing the premature failure of the bond [17] before the level of the retained bond is reached. The smaller the ratio of the concrete cover diameter to the bar diameter, the more prevalent this form of failure becomes. In the case of thicker concrete cover (approx. 4–5 times the bar diameter or a bar spacing more than 10 times the bar diameter), when the anchorage zone is provided with transverse reinforcement, or where compressive stresses act in a transverse direction (bar confinement), the bond failure is caused by pulling out the reinforcing bar as a result of the concrete wedges in spaces between the reinforcement ribs being sheared off (Figure 2a). Moreover, if the pulled-out bar is located at the outer edge of the reinforced concrete element and its concrete cover is relatively small, the bond failure may occur by the spalling of concrete cover, which is initiated by the splitting of the concrete cover [22]. Thus, in laboratory tests, the progress of the bond mechanism is determined by the choice of test type—e.g., pull-out test, beam-end test—requiring different sample types and cover thicknesses [23].

The splitting mode is the inferior of the two modes since the bond strength is restrained by the splitting resistance of the surrounding concrete cover and confining reinforcement, along with any transverse pressure [17]. The design rules are in general formulated on the weakest practical detailing arrangements consistent with other code provisions for minimum cover and bar spacing, etc., whereas models for local bond-slip behavior have generally been developed for high confinement conditions where splitting does not occur. However, since the majority of experimental programs have been based on highly confined samples, the investigated mechanisms in the paper are based on the pull-out tests.

Many researchers have attempted to formulate a uniform bond function, validated through experimentation, which would describe the failure mechanism in cracked elements in a simplified form [24,25,26,27,28,29]. Most of these efforts were based on the relationship between bond stresses and the displacement of the reinforcing bars against the concrete. These curves vary between modes of bond failure. In Model Code 2010 [30], they are additionally differentiated by confinement and bond conditions. The highest bond stresses in functions (*τ*_b,max_ for pull-out and *τ*_bu,slip_ for splitting) are typically estimated from the compressive strength of the concrete. Furthermore, bond strength is commonly calculated in relation to the concrete cover thickness and the reinforcing bar diameter.

## 3. Materials and Methods

### 3.1. Concrete Mixture

In this experiment, the authors produced fly ash-enriched SCC making use of the literature and their previous experience in the design of the mixture composition. Portland ash cement CEM II/B-V 32.5R (Gorazdze, Cracow, Poland) with a strength class of 32.5 N/mm^2^ and a high early strength was used. The cement met the requirements of EN 197-1:2012 [31]. Regarding the mineral addition, fly ash (FA) of category A and a fineness of category of N was used. Category A indicates that the loss on ignition is less than 5%. The total binder (cement and fly ash) share was taken to be 450 kg/m^3^, which is the lower limit recommended for SCC mixes. The water-to-binder ratio was set at 0.36. The percentage of fly ash in the total binder amount was fixed at 20%. The aggregates were in line with the requirements and categories expected within the normative guidelines [32]. Two fractions of gravel (coarse aggregate) of 2–8 mm and 8–16 mm were used. Natural sand with a fraction of 0–2 mm was used as a fine aggregate. The final aggregate density was 1400 kg/m^3^, and the sand point ratio was 50%. Polycarboxylic ether polymer superplasticizer was dosed in a way that ensured the proper fluidity and plastic viscosity of the mixture. The exact compositions of the mixes used in the experiment are given in Table 1.

### 3.2. Reinforcement Steel

**Figure 3:**
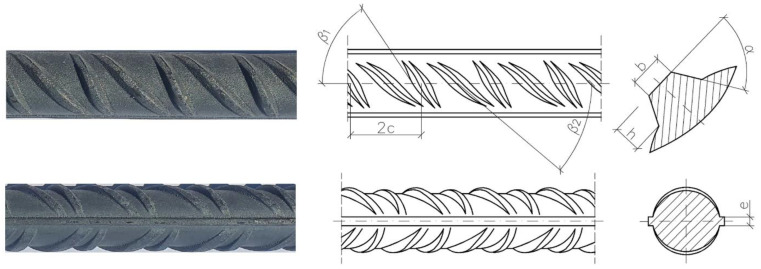
Geometry of B500SP reinforcing bars.

Ribbed reinforcing bars (B500SP) were used to test the bonds of the specimens described in Section 3.3. A representative diameter for the so-called mean diameters (10–20 mm) was used, which was 16 mm, in line with [24]. The geometric details of the pattern are given in Table 2. Figure 3 presents the characteristic rib pattern of reinforcing steel B500SP. There were two transverse ribs positioned alternately on both sides of a bar, typically accompanied by two ribs lengthwise.

### 3.3. Test Specimens and Basic Samples

**Figure 4:**
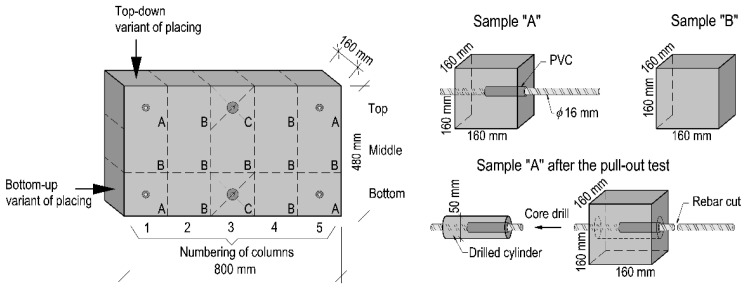
Schematic view of test panel elements and sample types.

The research focused on two 800 × 480 × 160 mm panel elements that were divisible into more basic 160 mm cubic modules, as shown in Figure 4. The module size is appropriate for pull-out tests with 16 mm bars, observing the EN 10080 [33] and RILEM TC [34] standards recommending that the size should be 10 d × 10 d × 10 d, where d is the bar diameter.

Elements were partitioned into columns labeled with numbers from 1 to 5. In the experiment, there was a single casting point positioned at column 1 at one edge of the element. Three types of basic module samples were extracted from the elements: samples A—for bond tests with embedded ribbed rebars, and samples B—for compressive strength tests. Concrete core samples with a diameter of 50 mm were taken from type A modules after the pull-out test and were used to visualize the steel–concrete interface by X-ray computed tomography scanner ‘phoenix v tome x m’ (General Electric, Boston, MA, USA) Type C specimens were not used in this study, since they were designated for an independent analysis.

In the experiment, the concrete placing was executed in two variants. The traditional approach (Variant I) assumed a placement from the top of the form downwards, whereas in the other scenario (Variant II), concrete was placed bottom-up. The latter variant was realized using piping fixed over the bottom level of the form and routed above the top level. In this variant, the concrete mixture arrived in the form and gravitationally filled it under its own weight from the bottom level upwards, as expected from the fundamental principle of connected vessels. When the bottom was fully flooded and the inlet level was reached, the incoming mixture (with a discharge rate of 0.5 l/s) lifted the fresh surface layer and facilitated its even distribution across the form. Two test panel elements were produced (one per variant).

After 3 days of curing, the formwork was stripped. The specimens underwent a curing process in fixed positions under laboratory conditions with continuous water sprinkling. Then, 3 weeks later, the panels were cut into smaller parts that subsequently, after 4 weeks, were subjected to strength and pull-out tests.

### 3.4. Test Procedures

#### 3.4.1. Test on Fresh Mixture

Fresh self-compacting mixes were tested in order to evaluate flow properties as well as air content. The experiments were conducted under identical laboratory conditions at an average temperature of 20 °C and average humidity of 50%. The flow properties of the fresh SCC were identified through three tests. Both flowability and fluidity [35] were estimated by the slump-flow test. The quantities measured were the final slump-flow diameter and the slump-flow time needed to reach a diameter of 500 mm, T_500_. The fresh visual segregation index specified in the slump-flow test was used to obtain the segregation resistance [36]. The L-box test, following [37], was conducted to find the passing ability. The flow tests were carried out immediately after mixing was complete.

Following the temporary stabilization of the mix in a standardized container, it was possible to find the air bubble content share using a pressure gauge as recommended by EN 12350-7 [38].

#### 3.4.2. Compressive Tests

The compressive strength was studied following the guidelines EN 12390-3:2009 [39] on cubic elements (approximately 160 × 160 × 160 mm) extracted from the test element marked as Sample B (Figure 4). This made it possible to observe the changes in the compressive strength of the specimens regarding the height and length. Furthermore, 10 150 mm cubic elements were cast and studied in terms of their compressive strength.

#### 3.4.3. Bond Strength Tests

In our experiment, out of several possibilities, the pull-out method was adopted to test the steel–concrete bond. This approach was in line with the recommendations of RILEM TC [34] and EN 10080 [33] and was carried out on cubic samples. This method is widely used for the examination of rebar–concrete interactions, which depend on the properties of the concrete and reinforcing bars. In such tests, a bar anchored in a concrete block experiences tensile load, leading to a displacement with respect to the material. Both the tensile load and displacement are measured to assess the initial bond.

The method assumes that the deformation along the bar is linear, and then the bond stress is approximated as constant and given by Equation (1):

(1) $$\tau =\frac{F}{\pi dL}$$

where *F*, *d,* and *L* correspond to the applied load, reinforcing bar diameter, and bond section length, respectively. The bond length—*L*—was experimentally taken to be 3.75 d. Adopting a higher value of *L*, such as the normative value of 5 d, would increase the bond forces so much that the reinforcing steel would yield, preventing pull-out failure. The required bond section length was achieved by a means of plastic tubes (PVC) inserted around the concrete-immersed part of the rebar.

Eight cubic modules were subjected to the pull-out test, where the load was exerted gradually up to a point of bond failure. The resultant slip of the unloaded rebar end was measured using two linear variable displacement transducers (LVDTs) operating in connection to a data collection piece of software.

In the literature, the authors of [4,6,40,41] used the so-called ultimate bond stress *τ*_max_ (bond strength) as an unambiguous quantity defined as the bond stress at the moment of bond failure. Another popular quantity [17,42] is the critical bond stress *τ*_0.25_ describing the rebar slip of 0.25 mm. Additionally, as advertised by RILEM TC [34] and various authors [4,43,44], one can follow Equation (2) and find *τ*_m_ the arithmetic mean on the bond stresses *τ*_0.01_, *τ*_0.10_, and *τ*_1.00_ associated with the slip values of 0.01, 0.10, and 1.00 mm, respectively.

(2) $$\tau_{m}=\frac{\tau_{0.01}+\tau_{0.10}+\tau_{1.00}}{3}$$

In the experiment, based on the relation between the bond stress and displacement, the slip at the time of *τ*_max_ was less than 1.00 mm. Thus, in an attempt to observe the RILEM guidelines [25] and follow Equation (2), the authors decided to replace *τ*_1.00_ with a more practical *τ*_0.5_ corresponding to a slip of 0.5 mm.

#### 3.4.4. X-ray Computed Tomography

**Figure 5:**
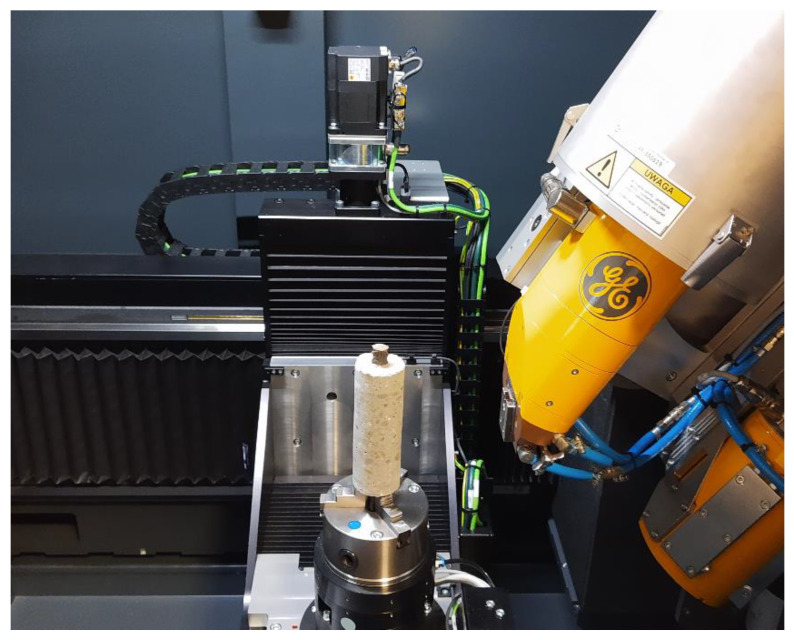
X-ray CT test set-up.

An X-ray computed tomography system was used to test specimens in order to verify the failure pattern of the sample after the pull-out test. The test setup is shown in Figure 5. A 300 kV mini focus lamp was used to emit X-rays with enough energy to penetrate the reinforced concrete sample and hit a detector. The visualization of the internal structure in 2D images with a resolution of 0.05 mm/pixel was possible through the detection of the density variation in the component and was inferred from the intensity of the incoming X-ray emission. During the CT scan, the specimen was rotated around its axis by 360° with a preset step of less than 1° and an X-ray source that remained still. In each position, digital 2D images were taken. Subsequently, the raw 2D images were combined into a tomographic 3D model in a process called reconstruction. The generated model was then used to find networks of tensile cracks around the rebars after the pull-out test. The analysis was performed using the Volume Graphics VGSTUDIO MAX software (Volume Graphics, Heidelberg, Germany).

## 4. Results

### 4.1. Fresh Mix Properties

Selected flow and air content properties of the fresh SCC used in the experiment are listed in Table 3. No segregation or bleeding effects were visible, meaning that the fresh visual stability index was 0. The air content of SCC mixes increased with the slump-flow and/or the plastic viscosity of the mix and was typically in the range of 2–5% [1], which is a higher level than in ordinary concretes.

### 4.2. Compressive Strength

Table 4 shows the results of the compressive strength tests performed on the samples. It is possible to compare the mean compressive strength of the concrete panels produced in the two placing variants. In the bottom-up case, the mean compressive strength was 51.6 MPa, whereas in the top-down scenario, it was 50.0 MPa. These values can be juxtaposed with that of the reference sample—47.3 MPa. Unfortunately, the low number of samples prevented us from studying the effect of the placing direction on the compressive strength in a statistically significant way. However, one could notice that samples located in the top part of the elements acquired the lowest values of compressive strength, regardless of the placing method.

### 4.3. Effect of Placing Direction on Bond Properties

**Figure 6:**
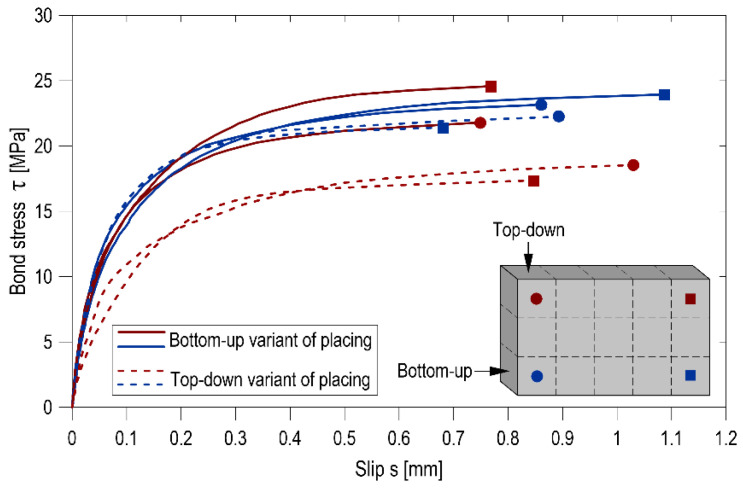
Bond stress vs. corresponding slip.

The relations between the bond stress and the slip of the rebar–concrete setup for both the top-down and bottom-up placing variants are visualized in Figure 6. The post-failure behaviour is not investigated in this study because it was assumed that the samples would be subjected to X-ray examination after pull-out testing. Table 5 provides the bond test results for the respectively ultimate (*τ*_max_), critical (*τ*_0.25_), and mean (*τ*_m_) normalized bond stresses. It is worth mentioning that all the samples exhibited pull-out bond failure.

The pull-out tests results show the influence of the SCC mix placing direction on the bond stresses, particularly in the top rebars. While for bottom rebars, this effect is ambiguous and does not significantly impact either of the representative bond stress values, for top rebars, an increase in the bond stresses was noted for those immersed in the elements concreted in a bottom-up method in comparison with a top-down placing variant. The lowest values of all the representative bond stresses in the tests were obtained for the top bars in the case of the top-down placing scenario. Depending on the selected representative stress, the bond stresses in the top bars of the element cast bottom-up were on average 29%, 33%, and 39% higher than those in the top bars of top-down cast elements for *τ*_max_, *τ*_0.25_, and *τ*_m_ respectively. As regards the bottom bars, the increase was only 8% for *τ*_max_, and for the other representative stresses, no significant differences were detected. Furthermore, a comparison of the representative bond stresses obtained for the top bars to the bottom bars indicated that the bottom-up placing of the mix resulted in a reduction of the top-bar effect.

An analysis of the bond–slip relationships led to the conclusion that their shape and slope result from the rebar position in the element and the direction of concrete placement. The placing technology did not affect the bond stiffness (a change in the bond strain increment relative to bar displacement in concrete) as far as the bottom rebars are concerned. However, the top rebars exhibited a strong enhancement of the bond stiffness when the concrete was placed bottom-up. The higher bond stiffness is linked with a higher quality of concrete underneath the ribs of the rebar; hence, the pull-out force that is needed to displace a section of the rebar must be greater.

### 4.4. Tomography Measurements

**Figure 7:**
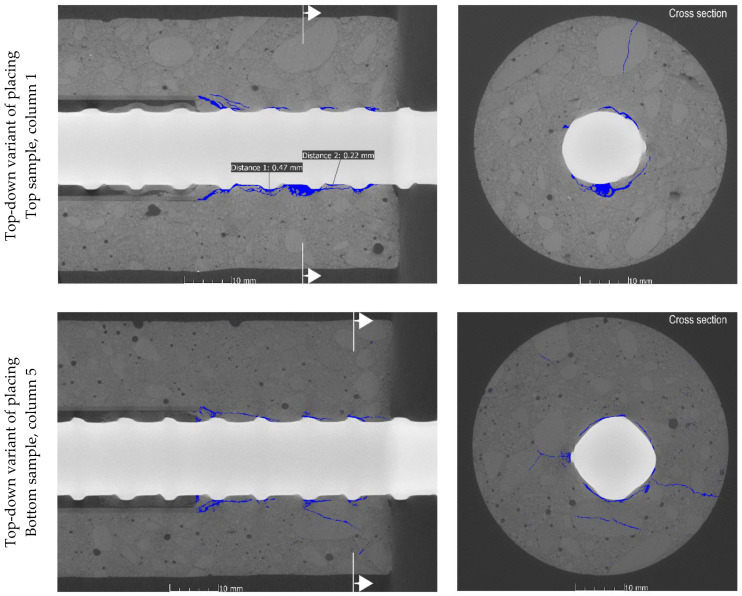

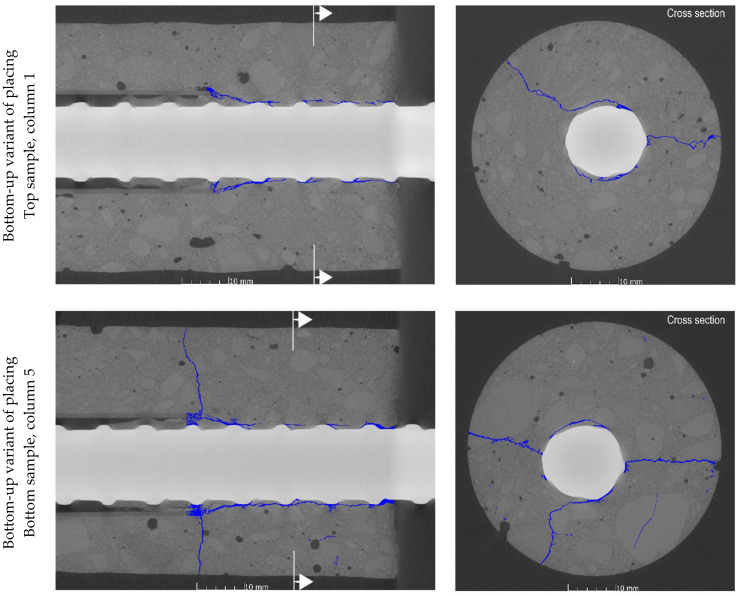
Examples of tomography sectioning of concrete cores extracted from the test panel.

Subsequent to the pull-out tests, cylindrical cores were extracted from the A specimens in order to perform the X-ray imaging of the steel–concrete interface. Using this imaging technique, it is possible to accurately assess cracks, porosity, and the presence of voids in the concrete specimens. The technique was focused on the area near the actual bond length of the rebar in order to detect the resulting structural damage of the concrete around the reinforcement. Furthermore, the limitation of the X-rayed material to a 50 mm diameter core was necessary to obtain high-quality and high-resolution images. However, during core drilling, the radial cracks occurring around the rebar (which, propagating to the surface, were withheld by the considerable cover thickness) caused the specimen to split in several cases. In the end, cylindrical cores from four specimens extracted from two different depths (bottom and top specimens) for the two SCC mix placement technologies were selected for X-ray CT and SCC behavior analysis after pullout testing (Figure 7).

**Figure 8:**
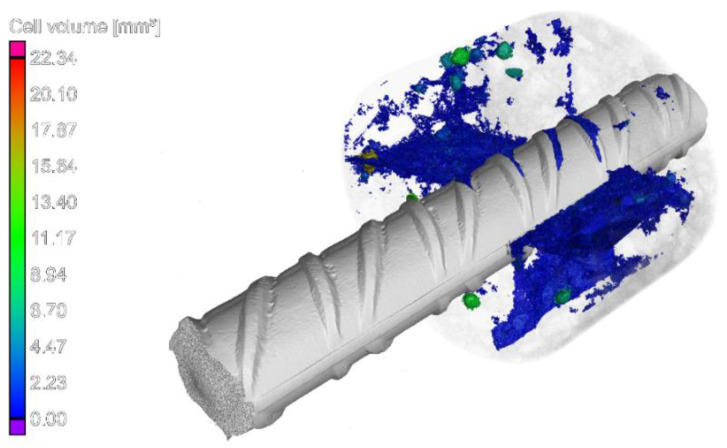
An example of the 3D reconstruction of X-ray CT scan (bottom-up variant of placement. Bottom sample, column 5).

The CT images represented a system of permanent structural discontinuities of the concrete as a result of crack formation associated with the action of the reinforcing steel-concrete joint. In the case of the top specimen extracted from the element made in traditional technology, additionally, the presence of significant air voids under the rebar was detected. SCI imaging performed after the pull-out test showed the presence of typical bond failure mechanisms as a result of pulling the rebar out of the concrete block. Regardless of the SCC placing method and rebar location, the formation of a mechanism of concrete surface shearing around the rebar in the distance corresponding to the height of the ribs in the form of a propagating crack parallel to the rebar axis (stage IVc of Figure 2) could be observed. The highest concentration and development of cracks was observed for each specimen directly under the rebar. The SCI imaging did not reveal the occurrence of transverse microcracks extending from the face of the ribs (stage II–III of bond failure in Figure 2). Due to the applied method of bond testing and thus the required cover thickness of the standard specimen and the subsequent bond failure mechanism, the potentially formed transverse microcracks could have been sealed as a result of the post-test release of the sample. It should also be noted that, with the exception of the top specimen collected from the element made in the traditional technology, the occurrence of radial–longitudinal cracks caused by concrete cover cracking was observed. These cracks propagated from the side end zone of the rib system (the location of the longitudinal overpressing of the rebar). An example of a three-dimensional reconstruction of the resultant cracking is presented in Figure 8.

For the top specimen collected from the panel element made with the top-down mix placing method, a void was observed directly under the reinforcing bar. The void spread along the bottom surface of the rebar and was caused by the bleeding and settlement of fresh concrete. It should be noted that the observed settlement of fresh concrete under the rebar was not regular. The most significant settlement and the largest voids formed primarily under the ribs, while smaller voids were formed in the space between them. A settlement of approximately 0.47 mm was found under the ribs and 0.22 mm between the ribs. Additionally, a trapped air bubble of significant volume (23.6 mm^3^) was noted under the rebar. For the other specimens, there was no fresh concrete settlement in the vicinity of the rebar. Consequently, the observed phenomena are reflected in the obtained pull-out test results.

### 4.5. Observation of Rebar–Concrete Interface after Splitting

**Figure 9:**
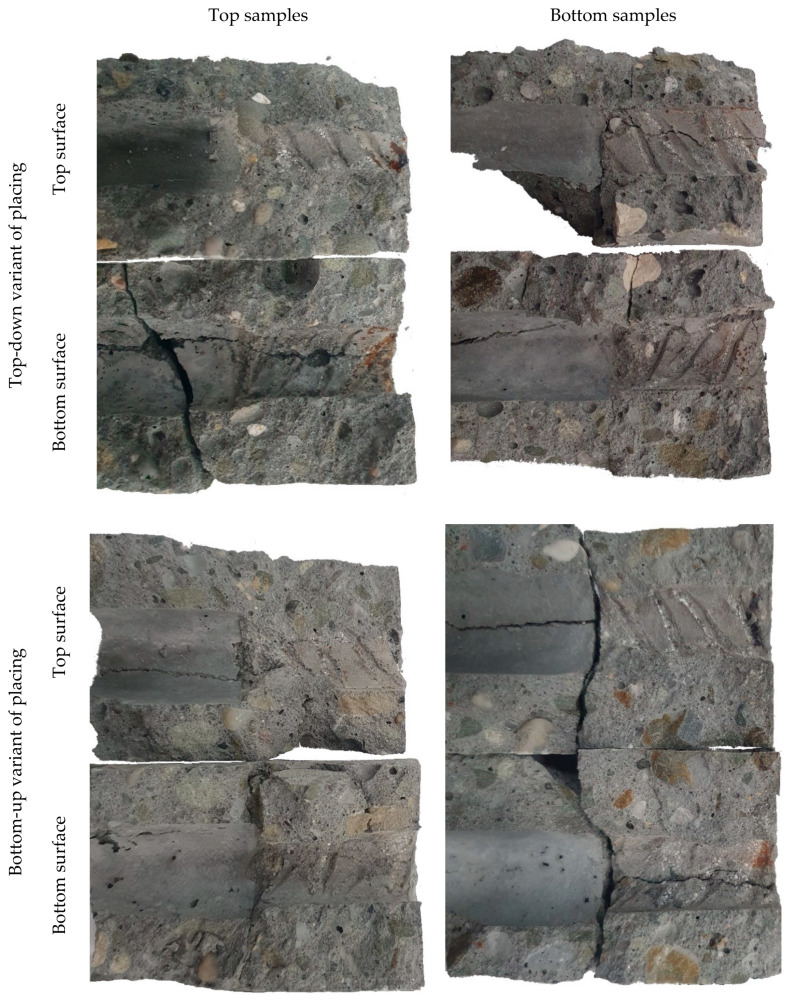
Views of SCC surface above and below rebar after pull-out tests.

In addition to non-destructive testing, to analyze the phenomena associated with the bond failure, the cylindrical cores of the A specimens were split after CT testing to inspect the condition of the reinforcing bar–concrete interface. The condition of the SCC around the rebar after the pull-out tests is presented in Figure 9. Regardless of the mixture placing method and the location of the specimens, clear rib marks were left on the concrete surface. Zones of concrete crushing by the front of the reinforcement ribs (stage III of bond failure of Figure 2) both above and below the rebars were visible; thus, the cooperation of materials in transferring bond forces occurred. In the case of the top specimen collected from the element made in traditional technology, the occurrence of a large air void and the settlement of the mix under the reinforcing bar were confirmed. These phenomena contributed to a decrease in the contact area between the bottom ribs of the rebar and the concrete. No deterioration of the concrete cover was observed in the remaining specimens.

## 5. Discussion

### 5.1. SCI Analysis Based on X-ray Computed Tomography

A visual inspection of the SCI zone using CT images showed that only a small area of the concrete structure damage is in contact with the steel surface. Therefore, after removing the rebars to expose the SCI, only a part of the resulting scratches is visible. Consequently, X-ray CT is more accurate and provides much better insight into the material than the visual inspection of the interface zone or scanning only selected cross-sections with a videomicroscope. Furthermore, when analyzing the concrete surface after splitting, radial–longitudinal cracks, which had propagated from the side ends of the rebar ribs (longitudinal overpressing location), remained undetected.

### 5.2. Effect of SCC Placing Method on Bond Failure

The morphology of the bond failure can be evaluated based on changes in the bond–slip relationship. The changes of this relation depend directly on the bond, which in turn is influenced by the condition of concrete in the vicinity of rebars. The second set of information related to the bond failure mechanism is the surface views of reinforcing bars and concrete in their surroundings after the pull-out test.

The examination of the bond–slip curves presented in Figure 6 indicates that their shape and slope depended on the vertical position of the rebars in the panel element and the method of concrete mix placement. In the case of the top-down placing of SCC, the bond stiffness of the top rebars is much lower than the bond stiffness of the bottom rebars. On the contrary, when the concrete was placed from the bottom to the top, the bond stiffness of the top rebars improved significantly (when compared to the top-down placing method situation) and equaled that of the bottom rebars. For the bottom rebars, the SCC placement method had no effect on the bond stiffness. This observation was confirmed by the obtained values of representative ultimate (*τ*_max_), critical (*τ*_0.25_), and mean (*τ*_m_) bond stresses presented in Table 5.

The main explanation for the decrease in the representative values of bond stresses as well as the bond stiffness of top specimens extracted from a panel element concreted from the top was the presence of voids under the rebar. Observations of the SCI zone indicated an effect between fresh concrete settlement and rebar, with two phenomena occurring. Firstly, the fresh concrete settled while the reinforcing bar remained attached to the formwork; secondly, the movement of the bleeding water was restrained by the reinforcement. The occurrence of voids under the rebar caused a reduction in the contact area of the bottom ribs of the rebar with the concrete or the exclusion of individual ribs from the transfer of pullout force. Furthermore, the properties of the concrete itself directly under the rebar deteriorated as a result of the bleeding phenomenon. These processes contributed to a non-uniform distribution of bond stresses along the bar axis and reduced bond stiffness. The bond failure was associated with the crushing of the concrete at the front of the bottom ribs and the development of longitudinal cracks, which formed at the tips of the ribs. This is a typical bond failure mechanism found in confined normal concretes, associated with the shearing of the concrete surface around the rebar at a distance corresponding to the height of the ribs [17].

During the study, it was noted that the bottom-up placement of SCC had a beneficial effect on the uniformity of bond quality across the vertical spans of the elements. The enhancement could be accounted for by the improvement of the contact surface quality in the top part of the panel element. The technology of placing SCC from the bottom of the form means that, at first, the injected mixture flows along the element until it reaches the level of the casting point opening. Then, the mix poured earlier gradually rises, and at the same time, it flows along the length of the element. The lifted layer of concrete is constantly self-ventilated and self-compacting during concrete works. In addition, in the bottom-up technology, there is no risk of introducing extra air into the mixture connected with the dropping of concrete. These observations were also confirmed by the studies [16]. CT images and the visual inspection of the surface after splitting clearly indicated that with the above-mentioned technology, the quality of SCC surrounding the rebar was significantly improved in comparison with the top-down placed concrete. This was reflected in an increase in the bond stress of the top rebars by an average of 29%, 33% and 39% for *τ*_max_, *τ*_0.25_, and *τ*_m_, respectively, and an increase in the bond stiffness. It should be noted that the same bond failure mechanism for the bottom and top bars was found for this technology. The homogeneous quality of the concrete cover around the rebar reduced the stress concentration to the rebar–concrete interface and increased the uniformity of the stress distribution. The formation of microcracks and their propagation are significantly reduced at lower loading levels. The observed bond failure was related to two known mechanisms: the development of longitudinal cracks, which formed at the tip of the ribs, and the exceeding of the tensile capacity in the concrete ring around the reinforcing bar [17,29]. The combination of these two failure mechanisms was due to the high compressive strength of concrete of 50 MPa. The high compressive strength of the concrete reduced the possibility of concrete crushing at the face of the ribs. The resulting high concentration of tensile stress in the concrete ring around the bar allowed longitudinal splitting cracks to appear. This crack developed radially outside from the periphery of the reinforcement and from the loading end until the free end of the rebar. The cracks propagated from the zone of the side end of the ribs (the location of the longitudinal overpressing of the rebar) but did not reach the outer surface of the cube specimen due to the large thickness of the cover.

*It should be noted that, regardless of the SCC placing technology, the highest concentration and development of longitudinal cracks caused by pull-out was observed directly under the reinforcing bar. This is due to the occurrence of concrete with lower strength properties directly under the horizontally placed rebars. This creates an inhomogeneous stress distribution in the reinforcing bar-concrete joint, which is not taken into account in the current models.*

[17,29,45]

### 5.3. Implication for Structural Concrete Design

Analysis of the SCI imaging using X-ray CT showed that, in reality, the formation of bond failure mechanisms is a complex process that depends on many factors. It was found that the described and known bond failure mechanisms were an idealized description of the actual operation of the rebar–concrete joint. The present investigations give additional information on the mechanisms of bond failure of horizontally positioned bars in the case of two different technologies of placing a SCC mixture: from the top and bottom of the form. It seems relevant to introduce into the standard recommendations and guidelines information about the occurrence of homogeneous mechanisms of a bond between reinforcing bars and concrete, irrespective of their position along the element height when the mix is applied from the bottom of the form. The uniformity of bond failure mechanisms along the height translates into uniform bond properties throughout the element: bond stiffness and bond strength, and thus in this case a reduced top bar effect. This may be potentially significant for special purpose structures that require increased durability or reliability, such as bridge structures, prestressed structures, etc. On the other hand, when it comes to normal structures, the demand for steel and thus costs could be reduced through the elimination of the need to increase the anchorage length and lap length in the zones with poor bond conditions.

## 6. Conclusions

The effect of two placement variants of self-compacting concrete– from the top and bottom of a formwork—on the bond failure mechanism has been studied experimentally in a comprehensive program, making use of the X-ray CT image analysis technique. The results and recommendations of this work are as follows:

- On the basis of the X-ray computed tomography analysis of cylindrical core samples collected from the top part of the panel element, it was discovered that the microstructure of the interface between the rebar and the concrete was fundamentally different between the two SCC placement methods under consideration, to the disadvantage of the traditional technology.
- The technology of placing concrete from the bottom of the formwork eliminated the occurrence of air voids and the settlement of the mix under the top rebar. Concrete cover exhibited improved quality, which increased the ultimate (*τ*_max_), critical (*τ*_0.25_), and mean (*τ*_m_) bond stresses by 29%, 33%, and 39%, respectively, in comparison to the mix application from the top.
- In the case of the bottom-up placement of SCC, the same mechanism of bond failure was observed for both bars located in the top and bottom part of the panel element. Thus, the bond properties of bond stiffness and bond strength and reduction of top-bar effect were the same across the whole element.
- *In the case of horizontally placed rebars, regardless of the SCC placing method, the highest concentration and development of longitudinal cracks were observed directly under the rebar. This caused an inhomogeneity of stress distribution in the reinforcing bar–concrete interface, which has not been considered in current failure modes*.
- The results provide valuable guidance for the design of reinforced concrete structures in order to achieve uniform conditions for the rebar–concrete joint throughout the member. Nevertheless, further investigations into this problem are needed.

## Figures and Tables

**Table 1:** Self-compacting concrete mix composition.

Composition [kg/m^3^]	SCC–FA20
Cement CEM II/B-V 32.5R	360
Water	160
Sand 0–2 mm	700
Gravel aggregate 2–8 mm	350
Gravel aggregate 8–16 mm	350
Fly ash	90
Superplasticizer	3.0
Binder content	450
Water/binder ratio	0.36
Fly ash level	20%

**Table 2:** Characteristics of surface and ribs of reinforcing bars (B500SP).

Parameter	Symbol	Units	Producer Limits	Measured Values
Flank inclination	α	degrees	≥45°	53°
Pitch angles	β1	degrees	35–75°	63°
	β2	degrees	35–75°	42°
Height	h	mm	0.03–0.15 d	1.4
Spacing	2c	mm	0.4–1.2 d	18.3
Width	b	mm	–	1.4
Longitudinal rebar width	e	mm	–	2.1

**Table 3:** Fresh properties test results.

Mix	Slump Flow [mm]	Slump Flow Class	Slump Flow Time T_50_ [s]	Viscosity Class	L-Box Ratio	L-Box Class	Fresh Visual Stability Index	Air Content [%]
SCC—FA20	690	SF2	1.8	VS1	0.91	PL2	0	2.4

**Table 4:** Compressive strength test results for cubic specimens.

Variant of Placing	Layer	Compressive Strength [MPa]						
		No. of Column (See Figure 4)					Mean	COV
		1	2	3	4	5		
Top-down	Top	A	47.9	C	47.0	A	47.5	0.9%
	Middle	52.4	50.4	51.3	53.2	52.6	52.0	2.0%
	Bottom	A	49.3	C	51.6	A	50.5	2.4%
Bottom-up	Top	A	48.2	C	49.9	A	49.1	1.8%
	Middle	52.2	52.6	53.3	55.9	53.5	53.5	3.4%
	Bottom	A	53.3	C	51.1	A	52.2	2.2%

A—sample used for pull-out test; C—sample used in another study [16]; COV—coefficient of variation.

**Table 5:** Respective bond stresses for the rebars in elements.

Variant of Placing	Layer	No. of Column (See Figure 4)					
		1			5		
		Ultimate Bond Stress *τ*_max_ [MPa]	Critical Bond Stress *τ*_0.25_ [MPa]	Mean Bond Stress *τ*_m_ [MPa]	Ultimate Bond Stress *τ*_max_ [MPa]	Critical Bond Stress *τ*_0.25_ [MPa]	Mean Bond Stress *τ*_m_ [MPa]
Top-down	Top	18.6	14.5	10.2	17.4	15.2	9.5
	Bottom	22.3	20.0	13.5	21.4	19.9	13.7
Bottom-up	Top	21.8	19.1	13.3	24.6	20.5	14.1
	Bottom	23.2	20.1	13.7	23.9	19.5	13.3

## Data Availability

The data that support the findings of this study are available from the corresponding author upon request.
